# Inadvertent radical nephrectomy leads to worse prognosis in renal pelvic urothelial carcinoma patients: A propensity score-matched study

**DOI:** 10.3389/fonc.2022.948223

**Published:** 2022-09-30

**Authors:** Feixiang Wu, Pan Zhang, Lingxun Li, Shiqing Lin, Jianhong Liu, Yi Sun, Yuanlong Wang, Chengjun Luo, Yu Huang, Xiao Yan, Meng Zhang, Guixi Liu, Kun Li

**Affiliations:** Department of Urology, The Third Hospital of Mianyang, Sichuan Mental Health Center, Mianyang, China

**Keywords:** renal pelvic urothelial carcinoma, survival, nephrectomy, nephroureterectomy, propensity score

## Abstract

**Objective:**

To compare overall survival (OS) and cancer-specific survival (CSS) in renal pelvic urothelial carcinoma (RPUC) patients treated with radical nephroureterectomy (NU) and inadvertent radical nephrectomy (RN).

**Patients and methods:**

In this retrospective study, patients with RPUC who underwent NU or RN diagnosed between 2004 and 2017 were identified from the Surveillance, Epidemiology, and End Results database. To adjust the confounders, the propensity score-matched analysis was conducted. The Kaplan–Meier method and log-rank test were performed to explore the effect of different surgical methods on OS and CSS.

**Results:**

A total of 2197 cases were finally included in this analysis, among which, 187 (8.5%) patients were treated with RN and 2010 (91.5%) patients were treated with NU. Before matching, the survival analysis revealed that the OS (HR: 1.444, 95%CI: 1.197, 1.741) and CSS (HR: 1.522, 95%CI: 1.211, 1.914) of patients who received RN were worse than that of patients who received NU (p = 0.0001 and p = 0.0003, respectively). After matching, the RN group had a worse OS (HR: 1.298, 95%CI: 1.002, 1.682) than the NU group (p = 0.048). No significant difference was observed in CSS between the RN and NU groups (p = 0.282). The hierarchical analysis showed that there was no significant difference observed in OS and CSS in patients with tumor size ≤4.2 cm (p = 0.884 and p = 0.496, respectively). In tumor size >4.2 cm, both OS (HR: 1.545, 95%CI: 1.225, 1.948) and CSS (HR: 1.607, 95%CI: 1.233, 2.095) of patients who received RN were worse than those of patients who received NU (p = 0.0002 and p = 0.0005).

**Conclusion:**

RN could lead to worse oncological outcomes than NU in patients with renal pelvis urothelial carcinoma. Accurate diagnosis of renal pelvis urothelial carcinoma is extremely important.

## Introduction

Upper tract urothelial carcinoma (UTUC) is composed of renal pelvic tumors and ureteral tumors and accounts for about 10% of kidney tumors (1–3). It is a low-incidence but aggressive malignancy with a poor prognosis. The incidence of renal pelvic tumors is approximately twice as that of ureteral tumors (3, 4). Besides, high-stage diseases (5, 6) are often observed in renal pelvic tumors at the time of first diagnosis. Regardless of the location of primary tumors, radical nephroureterectomy (NU) with bladder cuff resection is considered as the gold-standard treatment for non-metastatic UTUC (7–11). Conversely, in the management of renal cortical tumors, the types of surgery, including partial nephrectomy or radical nephrectomy (RN) with or without lymph node dissection, are selected depending on the location and tumor size of primary tumors (12).

The diagnosis of renal cancer, including both renal cortical tumor and renal pelvic tumor, relies heavily on abdominal imaging studies and invasive procedures such as ureteroscopy (13–15). Contrast-enhanced computed tomography and magnetic resonance imaging are the diagnostic mainstay for renal cancer (16). Due to its high sensitivity and specificity, renal biopsy prior to RN is not required for patients with imaging-diagnosed renal cell carcinoma. Sometimes, when a renal pelvis tumor infiltrates the renal cortex, it is difficult to differentiate intrarenal transitional cell carcinoma from centrally located renal cell carcinoma by imaging alone (17, 18). This phenomenon has resulted in some renal pelvic urothelial carcinomas (RPUC) being misdiagnosed as renal cell carcinomas, leading to the selection of RN when making the surgical decision. Currently, there are few reports on the misdiagnosis of intrarenal urothelial carcinoma as infiltrative renal cell carcinoma based on preoperative imaging (19). The impact of this error on the survival of patients with RPUC remains unclear. Some studies suggest that these changes in surgical management may lead to worse oncologic outcomes (20).

In this study, we recognized RPUC patients who were misdiagnosed as renal cell carcinoma and underwent RN from the Surveillance, Epidemiology, and End Results (SEER) database. Overall survival (OS) and cancer-specific survival (CSS) were compared in patients with RPUC who received RN and nephroureterectomy (NU) by adjusting for confounders using propensity score-matched (PSM) analysis.

## Patients and methods

### Population

Patients with pathologically confirmed RPUC who were diagnosed with renal cortical tumor (site code C64.9) and underwent RN between 2004 and 2017 were identified from the SEER database (Username: 10450-Nov2021). These patients were compared to patients with renal pelvic tumors (site code C65.9) and underwent NU.

The inclusion criteria were as follows: (1) patients with pathological diagnosis of RPUC, (2) age ≥18 years, (3) patients in stage M0, and (4) the histological subtypes of 8120/3, 8122/3, 8130/3, 8131/3. The exclusion criteria were (1) two or more primary tumors (2303) and (2) unknown tumor size (158).

### Data collection

The following clinical features were collected: age at diagnosis, year of diagnosis, marital status, sex, race, tumor size, laterality, grade, T, N, surgery of the primary tumor, systemic therapy, radiation, chemotherapy, survival time, and vital status. Age and tumor size were coded as continuous variables. For marital status, “divorced”, “separated”, “single”, “widowed”, “unmarried”, and “Unknown” were included in the “Unmarried/unknown” cohort. Race was divided into “White”, “Black”, and “Other” groups. Follow-up time for OS was calculated between diagnosis and death due to any cause, while CSS was calculated between diagnosis and death due to this cancer.

### Statistical analysis

Variables were reported with medians and interquartile ranges for continuous variables or frequencies and percentages for categorical variables. The differences between the two groups were analyzed using Mann–Whitney *U* and chi-square tests for continuous and categorical variables, respectively. To balance the confounders, we performed a 1:1 PSM analysis (logistic) between patients who underwent RN and NU, including age, marital status, sex, race, tumor size, laterality, grade, T, N, systemic therapy, radiation, and chemotherapy. The best cut-off value for tumor size was determined using X-tile based on survival status (21). The OS and CSS were analyzed using the Kaplan–Meier method and log-rank test. All statistical analyses were performed using SPSS 25.0 (IBM, Armonk, NY, USA) and R (version 4.1.1). Two-sided p < 0.05 was set as the cut-off criteria.

## Results

### Clinicopathological features

According to the inclusion and exclusion criteria, a total of 2197 cases were finally included in this analysis, among which, 187 (8.5%) patients were treated with RN and 2010 (91.5%) patients were treated with NU. All baseline characteristics are summarized in **Tables 1**, **2**. After the 1:1 PSM, in both the OS and CSS cohorts, patients who underwent RN had larger tumor size (both p < 0.001). Besides, year of diagnosis, race, N stage, and systemic therapy were also different between patients receiving RN and NU.

**Table 1:** The baseline demographic and clinicopathological features of patients with renal pelvic urothelial carcinoma in the overall survival cohort.

Variable^*^	Before match			After match		
	NU, N = 2,010*^1^*	RN, N = 187*^1^*	p-Value*^2^*	NU, N = 183*^1^*	RN, N = 183*^1^*	p-Value*^2^*
Size(cm)	3.6 (2.5, 5.1)	5.0 (3.5, 7.0)	<0.001	3.5 (2.5, 4.5)	5.0 (3.5, 7.0)	<0.001
Age(year)	71 (63, 79)	73 (63, 82)	0.3	70 (60, 78)	73 (63, 82)	0.048
Year of diagnosis			<0.001			<0.001
2004-2010	832 (41%)	103 (55%)		182 (99%)	100 (55%)	
2011-2017	1,178 (59%)	84 (45%)		1 (0.5%)	83 (45%)	
Sex			0.7			0.8
Female	919 (46%)	88 (47%)		83 (45%)	85 (46%)	
Male	1,091 (54%)	99 (53%)		100 (55%)	98 (54%)	
Race			0.11			0.014
White	1,734 (86%)	166 (89%)		149 (81%)	162 (89%)	
Black	97 (4.8%)	12 (6.4%)		9 (4.9%)	12 (6.6%)	
Other/unknown	179 (8.9%)	9 (4.8%)		25 (14%)	9 (4.9%)	
Marital status			0.9			0.8
Unmarried/unknown	819 (41%)	75 (40%)		73 (40%)	75 (41%)	
Married	1,191 (59%)	112 (60%)		110 (60%)	108 (59%)	
Laterality			0.2			0.3
Left	995 (50%)	101 (54%)		87 (48%)	98 (54%)	
Right	1,015 (50%)	86 (46%)		96 (52%)	85 (46%)	
Grade			0.14			<0.001
Low(G1-2)	306 (15%)	30 (16%)		59 (32%)	30 (16%)	
High(G3-4)	1,556 (77%)	136 (73%)		119 (65%)	132 (72%)	
Gx	148 (7.4%)	21 (11%)		5 (2.7%)	21 (11%)	
T			<0.001			0.001
T1	609 (30%)	64 (34%)		60 (33%)	64 (35%)	
T2	294 (15%)	9 (4.8%)		32 (17%)	9 (4.9%)	
T3	945 (47%)	86 (46%)		74 (40%)	83 (45%)	
T4	147 (7.3%)	28 (15%)		17 (9.3%)	27 (15%)	
Tx	15 (0.7%)	0 (0%)				
N			0.001			<0.001
N0	1,743 (87%)	147 (79%)		168 (92%)	146 (80%)	
N+	213 (11%)	37 (20%)		9 (4.9%)	34 (19%)	
Nx	54 (2.7%)	3 (1.6%)		6 (3.3%)	3 (1.6%)	
Systemic therapy			0.069			<0.001
No/unknown	1,609 (80%)	160 (86%)		178 (97%)	156 (85%)	
Yes	401 (20%)	27 (14%)		5 (2.7%)	27 (15%)	
Radiation			0.9			0.3
No/unknown	1,953 (97%)	182 (97%)		174 (95%)	178 (97%)	
Yes	57 (2.8%)	5 (2.7%)		9 (4.9%)	5 (2.7%)	
Chemotherapy			0.12			0.9
No/unknown	1,591 (79%)	157 (84%)		153 (84%)	154 (84%)	
Yes	419 (21%)	30 (16%)		30 (16%)	29 (16%)	

^1^Median (IQR); n (%).

^2^Wilcoxon rank sum test; Pearson’s Chi-squared test; Fisher’s exact test.

^*^ RN, radical nephrectomy; NU, nephroureterectomy.

**Table 2:** The baseline demographic and clinicopathological features of patients with renal pelvic urothelial carcinoma in the cancer-specific survival cohort.

Variable^*^	Before match			After match		
	NU, N = 1,668*^1^*	RN, N = 147*^1^*	p-Value*^2^*	NU, N = 143*^1^*	RN, N = 143*^1^*	p-Value*^2^*
Size(cm)	3.7 (2.5, 5.5)	5.5 (3.6, 7.0)	<0.001	3.5 (2.5, 4.4)	5.5 (3.6, 7.0)	<0.001
Age(year)	70 (62, 78)	69 (60, 80)	0.8	70 (61, 78)	69 (60, 80)	>0.9
Year of diagnosis			<0.001			<0.001
2004-2010	628 (38%)	79 (54%)		141 (99%)	76 (53%)	
2011-2017	1,040 (62%)	68 (46%)		2 (1.4%)	67 (47%)	
Sex			0.5			0.8
Female	758 (45%)	71 (48%)		66 (46%)	68 (48%)	
Male	910 (55%)	76 (52%)		77 (54%)	75 (52%)	
Race			0.033			<0.001
White	1,436 (86%)	128 (87%)		117 (82%)	124 (87%)	
Black	76 (4.6%)	12 (8.2%)		3 (2.1%)	12 (8.4%)	
Other/unknown	156 (9.4%)	7 (4.8%)		23 (16%)	7 (4.9%)	
Marital status			0.7			0.14
Unmarried/unknown	647 (39%)	59 (40%)		47 (33%)	59 (41%)	
Married	1,021 (61%)	88 (60%)		96 (67%)	84 (59%)	
Laterality			0.062			0.013
Left	819 (49%)	84 (57%)		60 (42%)	81 (57%)	
Right	849 (51%)	63 (43%)		83 (58%)	62 (43%)	
Grade			0.5			0.071
Low(G1-2)	239 (14%)	24 (16%)		35 (24%)	24 (17%)	
High(G3-4)	1,306 (78%)	109 (74%)		102 (71%)	105 (73%)	
Gx	123 (7.4%)	14 (9.5%)		6 (4.2%)	14 (9.8%)	
T			<0.001			0.054
T1	469 (28%)	46 (31%)		44 (31%)	46 (32%)	
T2	246 (15%)	7 (4.8%)		20 (14%)	7 (4.9%)	
T3	808 (48%)	69 (47%)		62 (43%)	66 (46%)	
T4	130 (7.8%)	25 (17%)		17 (12%)	24 (17%)	
Tx	15 (0.9%)	0 (0%)				
N			0.001			<0.001
N0	1,427 (86%)	111 (76%)		129 (90%)	110 (77%)	
N+	193 (12%)	33 (22%)		9 (6.3%)	30 (21%)	
Nx	48 (2.9%)	3 (2.0%)		5 (3.5%)	3 (2.1%)	
Systemic therapy			0.12			<0.001
No/unknown	1,293 (78%)	122 (83%)		136 (95%)	118 (83%)	
Yes	375 (22%)	25 (17%)		7 (4.9%)	25 (17%)	
Radiation			>0.9			0.2
No/unknown	1,617 (97%)	143 (97%)		134 (94%)	139 (97%)	
Yes	51 (3.1%)	4 (2.7%)		9 (6.3%)	4 (2.8%)	
Chemotherapy			0.2			0.9
No/unknown	1,278 (77%)	119 (81%)		115 (80%)	116 (81%)	
Yes	390 (23%)	28 (19%)		28 (20%)	27 (19%)	

^1^Median (IQR); n (%).

^2^Wilcoxon rank sum test; Pearson’s chi-squared test; Fisher’s exact test.

^*^RN, radical nephrectomy; NU, nephroureterectomy.

### Survival analysis

The median follow-up time was 42 (interquartile range 18–79) months for the entire cohort. Among all patients, 1177 (53.6%) died before the last follow-up, of which 795 (36.2%) patients died from RPUC.

**Figure 1:**
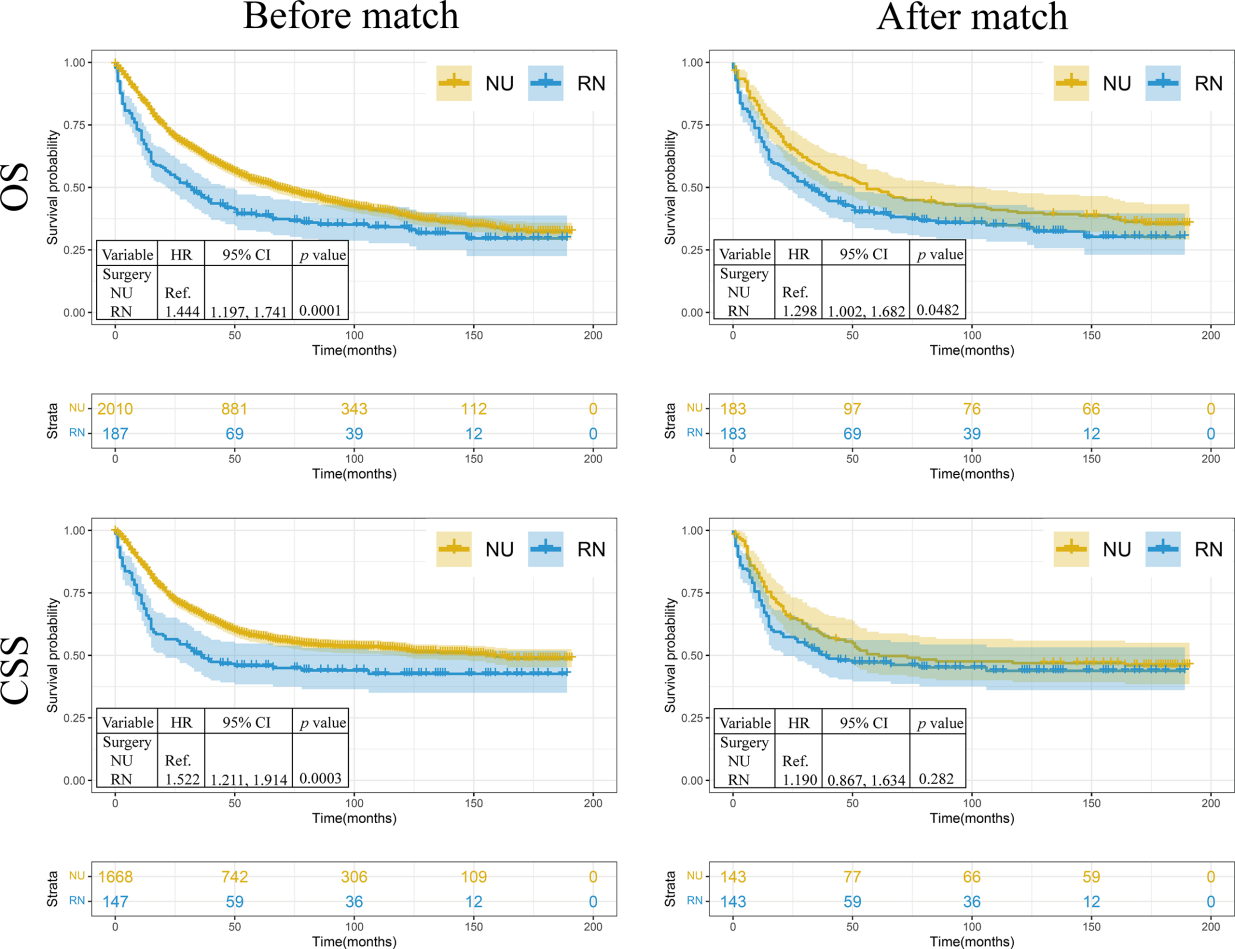
The Kaplan–Meier analysis of renal pelvic urothelial carcinoma treated with nephroureterectomy or radical nephrectomy. NU, nephroureterectomy; RN, radical nephrectomy; OS, overall survival; CSS, cancer-specific survival; HR, hazard ratio.

To compare prognostic differences between patients receiving PN and RN, the Kaplan–Meier method and log-rank test were performed. Before matching, the survival analysis revealed that the OS (HR: 1.444, 95%CI: 1.197, 1.741) and CSS (HR: 1.522, 95%CI: 1.211, 1.914) of patients who received RN were worse than those of patients who received NU (p = 0.0001 and p = 0.0003, respectively) (**Figure 1**). After matching, the RN group had worse OS (HR: 1.298, 95%CI: 1.002, 1.682) than the NU group (p = 0.048). No significant difference was observed in CSS (HR: 1.190, 95%CI: 0.8669, 1.634) between the RN and NU groups (p = 0.282) (**Figure 1**).

**Figure 2:**
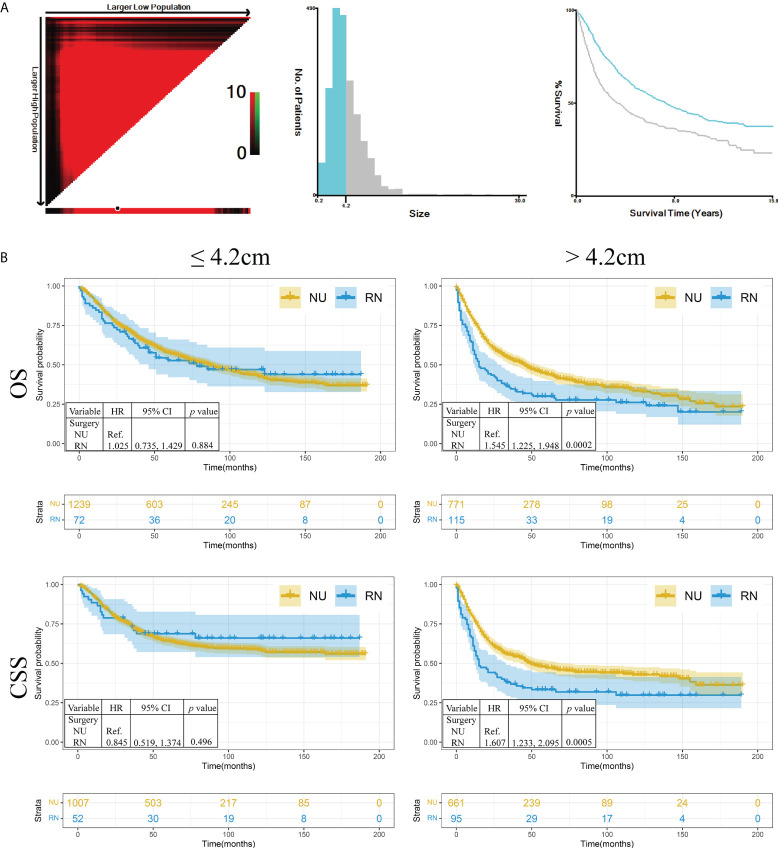
The hierarchical analysis of survival in different tumor sizes. **(A)** The tumor size was divided as two groups by X-tile. **(B)** The Kaplan–Meier analysis of renal pelvic urothelial carcinoma in different tumor sizes. NU, nephroureterectomy; RN, radical nephrectomy; OS, overall survival; CSS, cancer-specific survival; HR, hazard ratio.

To explore the role of RN and NU in patients with different tumor sizes, X-tile was performed to divide the entire cohort into small tumor size (≤4.2 cm) and large tumor size (>4.2 cm) groups (**Figure 2A**). The hierarchical analysis showed that no significant difference was observed in OS (HR: 1.025, 95%CI: 0.735, 1.429) and CSS (HR: 0.845, 95%CI: 0.519, 1.374) in patients with tumor size ≤4.2 cm (p = 0.884 and p = 0.496, respectively). In tumor size >4.2 cm, both OS (HR: 1.545, 95%CI: 1.225, 1.948) and CSS (HR: 1.607, 95%CI: 1.233, 2.095) of patients who received RN were worse than those of patients who received NU (p = 0.0002 and p = 0.0005, respectively) (**Figure 2B**).

## Discussion

In this retrospective study, we reported 187 patients (8.5%) with RPUC misdiagnosed as renal cell carcinoma who underwent RN between 2004 and 2017. In most cases, renal cancer is only assessed with abdominal imaging before surgery, and biopsy is not required. However, when kidney cancer infiltrates the renal pelvis or renal pelvic cancer infiltrates renal cortex, it is difficult to differentiate these two cancers by imaging alone, which leads to the misdiagnosis of renal pelvic urothelial carcinoma.

The summarized baseline characteristics showed that tumor size, year of diagnosis, race, N stage, and systemic therapy were different between patients who underwent RN and NU. Before PSM, both OS and CSS of patients treated with RN were worse than those of NU. After PSM, worse OS was observed in patients who underwent RN. No significant difference was observed in CSS between the RN and NU groups. The hierarchical analysis showed that worse OS and CSS were observed in patients who underwent RN with larger tumor size (>4.2 cm). The misdiagnosis of RPUC and the undergoing of RN also tend to occur in larger tumors due to similar renal masses (20). The worse oncologic outcomes observed in RPUC that underwent RN compared to that of NU cannot be accurately evaluated due to the present data. Some reasons could explain this disadvantage. The incomplete excision of the ureter and bladder cuff may increase the risk of tumor recurrence (22, 23). RPUC patients misdiagnosed as renal cortical tumor, even if postoperative pathology corrects the diagnosis, still results in missed opportunities for certain treatments, such as neoadjuvant chemotherapy, which can improve the outcomes of patients with high-risk or advanced RPUC (24–27). The standard extent of lymph node dissection for renal cortical tumors is not fully suitable for RPUC (28). Moreover, our study revealed that RN was related to worse survival especially in patients with larger tumors. Larger tumor size is associated with higher risk of muscle-invasive or non-organ-confined RPUC and higher risk of postoperative recurrence (29, 30). For smaller tumors, NU is also strongly recommended for better survival considering the multifocal nature of urothelial carcinoma, although no differences in OS and CSS were observed in this study.

The diagnosis of RPUC still mainly depends on computed tomography urography, ureteroscopy, and urine cytology (31). For both ureteroscopy and urine cytology, adequate samples are extremely important, considering variability and low sensitivity and specificity (32, 33). Considering the limitations of ureteroscopy in the diagnosis of muscle-invasive and high-grade RPUC, renal biopsy is recommended for the diagnosis of RPUC. These modalities can be combined to improve the accuracy of preoperative diagnosis. When conducting RN in suspected RPUC, the intraoperative frozen section may be helpful for the diagnosis (20). Most importantly, surgeons should be wary of urothelial carcinoma of the renal pelvis when performing RN in patients with renal cancer that has invaded the renal pelvis and has no pathological basis.

Some limitations of this study are as follows: 1. This is a retrospective study limited by its inherent bias. 2. The recurrence information was not included in the SEER database, leading to missing the recurrence-free survival. 3. The SEER database does not include information on preoperative hydronephrosis and surgical margin status. 4. The use of PS methods to adjust the confounders may lead to the exclusion of certain subsets that might influence the comparison. 5. There may be some variables or confounders that influence clinical decisions and outcomes that were not measured.

## Conclusion

Patients with RPUC misdiagnosed as renal cortical tumors treated with inadvertent RN had worse OS and CSS than patients treated with NU, especially in larger tumors.

## Data availability statement

Publicly available datasets were analyzed in this study. This data can be found here: SEER (https://seer.cancer.gov/).

## Ethics statement

Ethical review and approval was not required for the study on human participants in accordance with the local legislation and institutional requirements. Written informed consent for participation was not required for this study in accordance with the national legislation and the institutional requirements.

## Author contributions

All authors contributed to the design and conduct of the study. FW collected data and tissues, conducted data analysis, and wrote the manuscript. PZ, LL, SL, JL, YS, and YW contributed to design of the study and the collection of data. CL, YH, XY, MZ, GL, and KL participated in the collection of data and statistical analyses. All authors confirmed the final manuscript version.

## Acknowledgments

We are very grateful for the data sharing of the SEER database.

## Conflict of interest

The authors declare that the research was conducted in the absence of any commercial or financial relationships that could be construed as a potential conflict of interest.

## Publisher’s note

All claims expressed in this article are solely those of the authors and do not necessarily represent those of their affiliated organizations, or those of the publisher, the editors and the reviewers. Any product that may be evaluated in this article, or claim that may be made by its manufacturer, is not guaranteed or endorsed by the publisher.

## Abbreviations

RPUC, renal pelvic urothelial carcinoma; NU, nephroureterectomy; RN, radical nephrectomy; OS, overall survival; CSS, cancer-specific survival; SEER, Surveillance, Epidemiology, and End Results; PSM, propensity score-matched; UTUC, upper tract urothelial carcinoma; HR, hazard ratio; IQR, interquartile range.

## References

[1] Siegel RL Miller KD Fuchs HE Jemal A. Cancer statistics, 2021. CA Cancer J Clin (2021) 71(1):7–33. doi: 10.3322/caac.21654 33433946

[2] Chow WH Dong LM Devesa SS. Epidemiology and risk factors for kidney cancer. Nat Rev Urol (2010) 7(5):245–57. doi: 10.1038/nrurol.2010.46 PMC3012455 20448658

[3] Rouprêt M Babjuk M Burger M Capoun O Cohen D Compérat EM. European Association of urology guidelines on upper urinary tract urothelial carcinoma: 2020 update. Eur Urol (2021) 79(1):62–79. doi: 10.1016/j.eururo.2020.05.042 32593530

[4] Ouzzane A Colin P Xylinas E Pignot G Ariane MM Saint F. Ureteral and multifocal tumours have worse prognosis than renal pelvic tumours in urothelial carcinoma of the upper urinary tract treated by nephroureterectomy. Eur Urol (2011) 60(6):1258–65. doi: 10.1016/j.eururo.2011.05.049 21665356

[5] Yafi FA Novara G Shariat SF Gupta A Matsumoto K Walton TJ. Impact of tumour location versus multifocality in patients with upper tract urothelial carcinoma treated with nephroureterectomy and bladder cuff excision: a homogeneous series without perioperative chemotherapy. BJU Int (2012) 110(2 Pt 2):E7–13. doi: 10.1111/j.1464-410X.2011.10792.x 22177329

[6] Wang Q Zhang T Wu J Wen J Tao D Wan T. Prognosis and risk factors of patients with upper urinary tract urothelial carcinoma and postoperative recurrence of bladder cancer in central China. BMC Urol (2019) 19(1):24. doi: 10.1186/s12894-019-0457-5 30999871 PMC6471846

[7] Lughezzani G Burger M Margulis V Matin SF Novara G Roupret M. Prognostic factors in upper urinary tract urothelial carcinomas: A comprehensive review of the current literature. Eur Urol (2012) 62(1):100–14. doi: 10.1016/j.eururo.2012.02.030 22381168

[8] Soria F Shariat SF Lerner SP Fritsche HM Rink M Kassouf W. Epidemiology, diagnosis, preoperative evaluation and prognostic assessment of upper-tract urothelial carcinoma (UTUC). World J Urol (2017) 35(3):379–87. doi: 10.1007/s00345-016-1928-x 27604375

[9] Shvero A Hubosky SG. Management of upper tract urothelial carcinoma. Curr Oncol Rep (2022) 24(5):611–9. doi: 10.1007/s11912-021-01179-8 35212921

[10] Collà Ruvolo C Nocera L Stolzenbach LF Wenzel M Cucchiara V Tian Z. Incidence and survival rates of contemporary patients with invasive upper tract urothelial carcinoma. Eur Urol Oncol (2021) 4(5):792–801. doi: 10.1016/j.euo.2020.11.005 33293235

[11] Ryoo H Kim J Kim T Kang M Jeon HG Jeong BC. Effects of complete bladder cuff removal on oncological outcomes following radical nephroureterectomy for upper tract urothelial carcinoma. Cancer Res Treat (2021) 53(3):795–802. doi: 10.4143/crt.2020.919 33421984 PMC8291174

[12] Motzer RJ Jonasch E Agarwal N Bhayani S Bro WP Chang SS. Kidney cancer, version 2.2017, NCCN clinical practice guidelines in oncology. J Natl Compr Canc Netw (2017) 15(6):804–34. doi: 10.6004/jnccn.2017.0100 28596261

[13] Roberts JL Ghali F Aganovic L Bechis S Healy K Rivera-Sanfeliz G. Diagnosis, management, and follow-up of upper tract urothelial carcinoma: An interdisciplinary collaboration between urology and radiology. Abdom Radiol (NY) (2019) 44(12):3893–905. doi: 10.1007/s00261-019-02293-9 31701194

[14] Akita H Kikuchi E Hayakawa N Mikami S Sugiura H Oya M. Performance of diffusion-weighted MRI post-CT urography for the diagnosis of upper tract urothelial carcinoma: Comparison with selective urine cytology sampling. Clin Imaging (2018) 52:208–15. doi: 10.1016/j.clinimag.2018.08.012 30125847

[15] Rouprêt M Babjuk M Compérat E Zigeuner R Sylvester RJ Burger M. European Association of urology guidelines on upper urinary tract urothelial Carcinoma: 2017 update. Eur Urol (2018) 73(1):111–22. doi: 10.1016/j.eururo.2017.07.036 28867446

[16] Vogel C Ziegelmüller B Ljungberg B Bensalah K Bex A Canfield S. Imaging in suspected renal-cell carcinoma: Systematic review. Clin Genitourin Cancer (2019) 17(2):e345–55. doi: 10.1016/j.clgc.2018.07.024 30528378

[17] Raza SA Sohaib SA Sahdev A Bharwani N Heenan S Verma H. Centrally infiltrating renal masses on CT: differentiating intrarenal transitional cell carcinoma from centrally located renal cell carcinoma. AJR Am J Roentgenol (2012) 198(4):846–53. doi: 10.2214/AJR.11.7376 22451550

[18] Urban BA Buckley J Soyer P Scherrer A Fishman EK. CT appearance of transitional cell carcinoma of the renal pelvis: Part 2. Advanced-stage Disease AJR Am J Roentgenol (1997) 169(1):163–8. doi: 10.2214/ajr.169.1.9207518 9207518

[19] Ding X Ma X Jia Y Li H Wang Y. Intrarenal urothelial cancers confused as infiltrative renal masses: Report of 22 cases and literature review. Oncol Lett (2018) 16(2):1912–6. doi: 10.3892/ol.2018.8867 PMC6036454 30008883

[20] Al Hussein Al Awamlh B Shoag JE Basourakos SP Lewicki PJ Posada L Ma X. The consequences of inadvertent radical nephrectomy in the treatment of upper tract urothelial carcinoma. Urology (2021) 154:127–35. doi: 10.1016/j.urology.2021.03.003 33766715

[21] Camp RL Dolled-Filhart M Rimm DL. X-Tile: a new bio-informatics tool for biomarker assessment and outcome-based cut-point optimization. Clin Cancer Res (2004) 10(21):7252–9. doi: 10.1158/1078-0432.CCR-04-0713 15534099

[22] Lughezzani G Sun M Perrotte P Shariat SF Jeldres C Budaus L. Should bladder cuff excision remain the standard of care at nephroureterectomy in patients with urothelial carcinoma of the renal pelvis? a population-based study. Eur Urol (2010) 57(6):956–62. doi: 10.1016/j.eururo.2009.12.001 20018438

[23] Xylinas E Rink M Cha EK Clozel T Lee RK Fajkovic H. Impact of distal ureter management on oncologic outcomes following radical nephroureterectomy for upper tract urothelial carcinoma. Eur Urol (2014) 65(1):210–7. doi: 10.1016/j.eururo.2012.04.052 22579047

[24] Porten S Siefker-Radtke AO Xiao L Margulis V Kamat AM Wood CG. Neoadjuvant chemotherapy improves survival of patients with upper tract urothelial carcinoma. Cancer (2014) 120(12):1794–9. doi: 10.1002/cncr.28655 PMC4440575 24633966

[25] Chen L Ou Z Wang R Zhang M He W Zhang J. Neoadjuvant chemotherapy benefits survival in high-grade upper tract urothelial carcinoma: A propensity score-based analysis. Ann Surg Oncol (2020) 27(4):1297–303. doi: 10.1245/s10434-019-08128-7 31853757

[26] Seisen T Colin P Hupertan V Yates DR Xylinas E Nison L. Postoperative nomogram to predict cancer-specific survival after radical nephroureterectomy in patients with localised and/or locally advanced upper tract urothelial carcinoma without metastasis. BJU Int (2014) 114(5):733–40. doi: 10.1111/bju.12631 24447471

[27] Matsunaga T Komura K Hashimoto T Muraoka R Satake N Tsutsumi T. Adjuvant chemotherapy improves overall survival in patients with localized upper tract urothelial carcinoma harboring pathologic vascular invasion: a propensity score-matched analysis of multi-institutional cohort. World J Urol (2020) 38(12):3183–90. doi: 10.1007/s00345-020-03118-x 32065276

[28] Roscigno M Brausi M Heidenreich A Lotan Y Margulis V Shariat SF. Lymphadenectomy at the time of nephroureterectomy for upper tract urothelial cancer. Eur Urol (2011) 60(4):776–83. doi: 10.1016/j.eururo.2011.07.009 21798659

[29] Collà Ruvolo C Nocera L Stolzenbach LF Wenzel M Califano G Tian Z. Tumor size predicts muscle-invasive and non-organ-confined disease in upper tract urothelial carcinoma at radical nephroureterectomy. Eur Urol Focus (2022) 8(2):498-505. doi: 10.1016/j.euf.2021.03.003 33737024

[30] Espiritu PN Sverrisson EF Sexton WJ Pow-Sang JM Poch MA Dhillon J. Effect of tumor size on recurrence-free survival of upper tract urothelial carcinoma following surgical resection. Urol Oncol (2014) 32(5):619–24. doi: 10.1016/j.urolonc.2013.11.006 24495448

[31] Territo A Foerster B Shariat SF Rouprêt M Gaya JM Palou J. Diagnosis and kidney-sparing treatments for upper tract urothelial carcinoma: State of the art. Minerva Urol Nefrol (2018) 70(3):242–51. doi: 10.23736/S0393-2249.18.03058-8 29392926

[32] Smith AK Stephenson AJ Lane BR Larson BT Thomas AA Gong MC. Inadequacy of biopsy for diagnosis of upper tract urothelial carcinoma: implications for conservative management. Urology (2011) 78(1):82–6. doi: 10.1016/j.urology.2011.02.038 21550642

[33] Messer J Shariat SF Brien JC Herman MP Ng CK Scherr DS. Urinary cytology has a poor performance for predicting invasive or high-grade upper-tract urothelial carcinoma. BJU Int (2011) 108(5):701–5. doi: 10.1111/j.1464-410X.2010.09899.x 21320275

